# (GAA)n microsatellite as an indicator of the A genome reorganization during wheat evolution and domestication

**DOI:** 10.3897/CompCytogen.v9i4.5120

**Published:** 2015-09-02

**Authors:** Irina G. Adonina, Nikolay P. Goncharov, Ekaterina D. Badaeva, Ekaterina M. Sergeeva, Nadezhda V. Petrash, Elena A. Salina

**Affiliations:** 1 Institute of Cytology and Genetics, Siberian Branch, Russian Academy of Sciences, pr. Lavrentieva 10, Novosibirsk, 630090 Russia; 2 N.I.Vavilov Institute of General Genetics, Russian Academy of Sciences, Gubkina street 3, Moscow 119991, Russia; 3 Siberian Research Institute of Plant Growing and Selection – Branch of ICG SB RAS, Krasnoobsk, Novosibirsk Region, Russia

**Keywords:** *Triticum monococcum*, *Triticum boeoticum*, *Triticum urartu*, *Triticum zhukovskyi*, *Triticum dicoccoides*, (GAA)n microsatellite, FISH

## Abstract

Although the wheat A genomes have been intensively studied over past decades, many questions concerning the mechanisms of their divergence and evolution still remain unsolved. In the present study we performed comparative analysis of the A genome chromosomes in diploid (*Triticum urartu* Tumanian ex Gandilyan, 1972, *Triticum boeoticum* Boissier, 1874 and *Triticum monococcum* Linnaeus, 1753) and polyploid wheat species representing two evolutionary lineages, Timopheevi (*Triticum timopheevii* (Zhukovsky) Zhukovsky, 1934 and *Triticum zhukovskyi* Menabde & Ericzjan, 1960) and Emmer (*Triticum dicoccoides* (Körnicke ex Ascherson & Graebner) Schweinfurth, 1908, *Triticum durum* Desfontaines, 1798, and *Triticum aestivum* Linnaeus, 1753) using a new cytogenetic marker – the pTm30 probe cloned from *Triticum monococcum* genome and containing (GAA)_56 _microsatellite sequence. Up to four pTm30 sites located on 1AS, 5AS, 2AS, and 4AL chromosomes have been revealed in the wild diploid species, although most accessions contained one–two (GAA)n sites. The domesticated diploid species *Triticum monococcum* differs from the wild diploid species by almost complete lack of polymorphism in the distribution of (GAA)n site. Only one (GAA)n site in the 4AL chromosome has been found in *Triticum monococcum*. Among three wild emmer (*Triticum dicoccoides*) accessions we detected 4 conserved and 9 polymorphic (GAA)n sites in the A genome. The (GAA)n loci on chromosomes 2AS, 4AL, and 5AL found in of *Triticum dicoccoides* were retained in *Triticum durum* and *Triticum aestivum*. In species of the Timopheevi lineage, the only one, large (GAA)n site has been detected in the short arm of 6A^t^ chromosome. (GAA)n site observed in *Triticum monococcum* are undetectable in the A^b^ genome of *Triticum zhukovskyi*, this site could be eliminated over the course of amphiploidization, while the species was established. We also demonstrated that changes in the distribution of (GAA)n sequence on the A-genome chromosomes of diploid and polyploid wheats are associated with chromosomal rearrangements/ modifications, involving mainly the NOR (nucleolus organizer region)-bearing chromosomes, that took place during the evolution of wild and domesticated species.

## Introduction

The genus *Triticum* Linnaeus, 1753 comprises species at different ploidy levels, from diploid to hexaploid. Common wheat *Triticum aestivum* L., 1753 is natural allopolyploid with the genome BBAADD, which emerged about 8–10 thousand years ago (TYA) *via* the cross of tetraploid Emmer species (BBAA genome) with *Aegilops tauschii* Cosson, 1850 (DD genome). Another hexaploid wheat, *Triticum zhukovskyi* Menabde & Ericzjan, 1960 (genome GGA^t^A^t^A^b^A^b^) was discovered in 1957, in the Zanduri region of Western Georgia and is regarded as natural allopolyploid of *Triticum timopheevii* (Zhukovsky) Zhukovsky, 1934 and *Triticum monococcum* L., 1753 growing in the same area (Jakubtsiner 1959, Tavrin 1964). As is currently assumed by the majority of researchers, tetraploid Emmer (*Triticum dicoccoides* ((Körnicke ex Ascherson & Graebner) Schweinfurth, 1908, *Triticum durum* Desfontaines, 1798, etc. genome BBAA) and Timopheevi (*Triticum araraticum* Jakubziner, 1947, *Triticum timopheevii*, and *Triticum militinae* Zhukovsky & Miguschova, 1969, genome GGA^t^A^t^) wheats occurred as a result of hybridization between the ancestral forms of *Aegilops speltoides* Tausch, 1837 as a maternal parent and *Triticum urartu* Tumanian ex Gandilyan, 1972, as a paternal parent (Dvorak et al. 1988, Tsunewaki 1996, Huang et al. 2002). Although both evolutionary lineages of the tetraploid wheats originated *via* hybridization of closely related parental forms, their emergence occurred independently at different times and probably in different places. In particular, the origin of the tetraploid *Triticum dicoccoides* is dated back to over 500 TYA, versus *Triticum araraticum*, dated back to 50–300 TYA (Mori et al. 1995, Huang et al. 2002, Levy and Feldman 2002).

The diploid wheats are the most ancient members of the genus *Triticum*. Among them taxonomists recognize three species, namely, cultivated *Triticum monococcum* and two wild species, *Triticum boeoticum* Boissier, 1874 and *Triticum urartu* (Goncharov, 2012). Two different types of the A genome, A^u^ (*Triticum urartu*) and A^b^ (*Triticum boeoticum* and *Triticum monococcum* L., 1753), have been discriminated among the diploid wheats. According to the current concept, the A^u^ and A^b^ genomes diverged approximately one million years ago (Huang et al. 2002). Morphologically *Triticum urartu* and *Triticum boeoticum* are very similar (Filatenko et al. 2002), and differ distinctly only in the leaf pubescence pattern (velvety *vs.* bristly), controlled by allelic genes (Golovnina et al. 2009). These wild species have overlapping distribution ranges, and in some cases accessions belonging to either one of the species are identified incorrectly.

Despite morphological similarity, the level of genome divergence between *Triticum urartu* and *Triticum boeoticum* is very high. First of all it is indicated by the sterility of hybrids between *Triticum urartu* and *Triticum boeoticum* and/or *Triticum monococcum*, although in certain combinations of accessions and crossing direction hybrid fertility was elevated from zero to 4.5% (Fricano et al. 2014). Analysis of a broad sample of diploid A-genome species using multilocus markers, such as SSAP (sequence specific amplification polymorphism) and AFLP (amplified fragment length polymorphism) demonstrated considerable genetic differentiation among the accessions; and two super-clusters of diploid wheats have been discriminated among them (Konovalov et al. 2010, Fricano et al. 2014). The first super-cluster contains *Triticum urartu* and the second one, domesticated species *Triticum monococcum* and its wild progenitor, *Triticum boeoticum*. Importantly, intermediate forms between two super-clusters are detectable independently of the approach used for analysis; moreover, solitary accessions morphologically affiliated with *Triticum urartu* fall either within the opposite cluster or close to it.

Genome rearrangements, such as translocations, inversions, and the emergence of large blocks of repeats *via* amplification, are of considerable importance for the reproductive isolation of species. Such large-scale rearrangements are detectable by meiotic chromosome pairing analysis, comparative genome mapping, and FISH with repetitive probes. The data obtained so far suggest that the emergence of two evolutionary lineages of polyploid wheats, Emmer and Timopheevi, was accompanied by several species-specific translocations (Rodriguez et al. 2000, Salina et al. 2006a). Only one of these translocations, 4AL/5AL, which was inherited by polyploid wheat species from their diploid A genome progenitor, is characteristic of both *Triticum urartu* and *Triticum monococcum* (King et al. 1994, Devos et al. 1995). No information concerning the detection of other intraspecific and interspecific translocations in diploid wheats is available from literature.

One of the approaches for the identification of chromosomal rearrangements is cytogenetic analysis. Despite significant progress in sequencing and mapping of cereal genomes, this method is still most powerful for detection of chromosome aberrations; however, it needs a sufficient pool of cytogenetic markers. The number of cytogenetic markers used for the analysis of A genome chromosome is currently rather few. Single hybridization signals can the obtained with probes pSc119.2, pAs1, pTa71 (45S RNA genes), and pTa794 (5S rRNA genes) (Dubcovsky and Dvořák 1995, Schneider et al. 2003, Megyeri et al. 2012, Uhrin et al. 2012). Several (GAA)n sites have been detected on the A genome chromosomes of polyploid wheat, although signals are located predominantly on the B and G genome chromosomes of wheats and in the S-genome chromosomes of their diploid progenitor *Aegilops speltoides* (Gerlach and Dyer 1980). Hybridization with the (GAA)n probe not always produces stable signals on the A genome chromosomes, since either a synthetic probe or PCR fragments amplified from the wheat or rye genomic DNA were used (Kubaláková et al. 2005, Megyeri et al. 2012).

The goal of this work was to study the rearrangement of the A genome chromosomes of wheats during the evolution based on the distribution of (GAA)n microsatellite on the chromosomes.

## Materials and methods

### Plant material

The following diploid Triticum species were used in our work (see Table 1 for the complete list): *Triticum boeoticum* (2n = 2x = 14, A^b^A^b^) – six accessions; *Triticum monococcum* (2n = 2x = 14, A^b^A^b^) – six accessions, and *Triticum urartu* (2n = 2x = 14, A^u^A^u^) – seven accessions. For each species we selected accessions differing in the level of genomic divergence. In particular, according to SSAP analysis based on the *BARE-1* and *Jeli* retrotransposons (Konovalov et al. 2010), all accessions of diploid wheats were divided into groups and super-clusters (Table 1). Current analysis included accessions from both well-differentiated super-clusters, *Triticum urartu* and *Triticum boeoticum*/*Triticum monococcum*, as well as three *Triticum urartu* accessions (UR3,UR4,UR5) for which the genome affinity determined by morphological traits was not confirmed by molecular analysis (Konovalov et al. 2010).

**Table 1.** Accessions of the diploid and polyploid Triticum species used in the work.

Accession/ ^*^group	Species	Subspecies/variety (if available)	Centre of genetic resource	Accession number	Geographic origin
BO2/IG1	*Triticum boeoticum*	subsp. thaoudar	Kyoto Univ.	KU8120	Iraq
BO3/IG2	*Triticum boeoticum*	–	VIR	K-25811	Armenia
BO9/IG1	*Triticum boeoticum*	–	ICARDA	IG116198	Turkey
BO12/IG2	*Triticum boeoticum*	subsp. boeoticum	VIR	K-18424	Crimea
BO14/IG1	*Triticum boeoticum*	–	USDA	PI427328	Iraq
BO19/IG2	*Triticum boeoticum*	subsp. boeoticum	VIR	K-33869a	Armenia
MO1/IG3	*Triticum monococcum*	var. macedonicum	VIR	K-18140	Azerbaijan
MO3/IG3	*Triticum monococcum*	var. monococcum	VIR	K-20409	Spain
	*Triticum monococcum*	–	VIR	K-18105	Nagorno-Karabakh Autonomous Region
	*Triticum monococcum*	–	VIR	K-8555	Crimea
	*Triticum monococcum*	–	USDA	PI119423	Turkey
	*Triticum monococcum*	var. hornemannii, population Zanduri	VIR	K-46586	Georgia
UR1/IIG4	*Triticum urartu*	–	USDA	PI538736	Lebanon
UR2/IIG4	*Triticum urartu*	var. albinigricans	VIR	K-33869b	Armenia
UR3/IG3	*Triticum urartu*	–	USDA	PI428276	Lebanon
UR4/IG1	*Triticum urartu*	–	ICARDA	IG116196	Turkey
UR5/IG2	*Triticum urartu*	var. albinigricans	VIR	K-33871	Armenia
UR6/IIG4	*Triticum urartu*	–	ICARDA	IG45298	Syria
UR44/IIG4	*Triticum urartu*	–	USDA	PI428182	Armenia
	*Triticum timopheevii*	population Zanduri	VIR	K-38555	Georgia
	*Triticum zhukovskyi*	population Zanduri	VIR	K-43063	Georgia
	*Triticum dicoccoides*		ICARDA	IG46273	Israel
	*Triticum dicoccoides*		ICARDA	IG46288	Israel
	*Triticum dicoccoides*		ICARDA	IG139189	Jordan
	*Triticum durum*		VIR	K-1931	Russia
	*Triticum aestivum*		ICG	cv. Chinese Spring	China
	*Triticum aestivum*	var. lutescens	ICG	cv. Saratovskaya 29	Russia

* Designation of accessions and their clustering into groups (I/II, superclusters and G, groups) are according to Konovalov et al. (2010).

Polyploid wheat species belonging to either Timopheevi (*Triticum timopheevii*, 2n = 4x = 28, GGA^t^A^t^, and *Triticum zhukovskyi*, 2n = 6x = 42, GGA^t^A^t^A^b^A^b^), or Emmer evolutionary lineage (*Triticum dicoccoides*, 2n = 4x = 28, BBAA, *Triticum durum*, 2n = 4x = 28, BBAA, and *Triticum aestivum*, 2n = 6x= 42, BBAADD) were analyzed (Table 1).

The plants of all accessions used in our work were grown at the Joint Access Laboratory for Artificial Plant Cultivation for verification of species authenticity by morphological characters using a guide published by Goncharov (2012).

*Triticum zhukovskyi* authenticity was verified by electrophoresis of wheat storage proteins (gliadins) (Goncharov et al. 2007).

### DNA isolation and cloning

The (GAA)n microsatellite sequence was cloned from einkorn wheat genome in order to increase the resolution of FISH analysis.

Total DNA was isolated from 5–7-day-old seedlings according to Plaschke et al. (1995). PCR for production of (GAA)n microsatellite was conducted according to Vrána et al. (2000) using (CTT)_7_ and (GAA)_7_ as primers and *Triticum urartu* (IG45298) and *Triticum monococcum* (PI119423) DNAs as templates. PCR comprised 35 cycles of denaturation at 94 °C for 1 min, annealing at 55 °C for 1 min, and synthesis at 72 °C for 1 min. The amplification products were cloned with a Qiagen kit. The clones differing in the length of the insert were selected and sequenced using ABI PRISM Dye Terminator Cycle Sequencing ready reaction kit (Perkin Elmer Cetus, USA). Sequencing was performed in an ABI PRISM 310 Genetic Analyzer (Perkin Elmer Cetus).

### Giemsa C-banding

Giemsa C-banding was performed according to the protocol by Badaeva et al. (1994). The slides were examined with a Leitz Wetzlar microscope and recorded with a Leica DFC 280 CCD digital camera. The chromosomes were classified according to the standard nomenclature (Friebe and Gill 1996, Gill et al. 1991).

The work was performed at the Vavilov Institute of General Genetics, Russian Academy of Sciences.

### Fluorescence *in situ* hybridization

Fluorescence *in situ* hybridization (FISH) was conducted as earlier described (Salina et al. 2006b). The probes were labeled with biotin or digoxigenin by nick translation. Biotinylated probes were detected with fluorescein avidin D (Vector Laboratories). The digoxigenin-labeled probes were detected with antibodies to anti-digoxigenin-rhodamine, Fab fragments (Roche Applied Science).

The chromosomes were identified using the pSc119.2 (120 bp, rye repeats; Bedbrook et al. 1980) or pTa71 (45S RNA genes; Gerlach and Bedbrook 1979) probes.

The preparations were embedded into Vectashield mounting medium (Vector Laboratories), containing 0.5 µg/ml DAPI (4’,6-diamidino-2-phenylindole, Sigma) for chromosome staining. The chromosomes were examined with an Axioskop 2 Plus (Zeiss) microscope and recorded with a VC-44 (PCO) CCD camera.

The work was performed at the Joint Access Center for Microscopic Analysis of Biological Objects with the Siberian Branch of the Russian Academy of Sciences.

## Results

### Cloning of (GAA)n microsatellite

Totally, four clones differing in the length of the insert were selected and sequenced. The clones obtained from *Triticum urartu* were designated pTu and from *Triticum monococcum*, pTm. All the clones contain (GAA)n microsatellite sequence, but differ in length: pTm30 has a length of 167 bp [(GAA)_56_]; pTm17, 62 bp [(GAA)_21_]; pTu33, 56 bp [(GAA)_19_]; and pTu38, 36 bp [(GAA)_12_]. The (GAA)_56_ microsatellite variant pTm30 generating most distinct signals was selected for further work.

### Localization of (GAA)n probe on einkorn wheat chromosomes

The new probe pTm30 containing (GAA)_56_ sequence was hybridized to chromosomal preparations of *Triticum urartu*, *Triticum boeoticum* and *Triticum monococcum*; the pTa71 (45S rRNA genes) probe was used for chromosome identification. As was expected, the 45S ribosomal RNA genes in all species were localized to the nucleolar organizer region in the distal parts of the 1AS and 5AS chromosomes.

**Figure 1.**
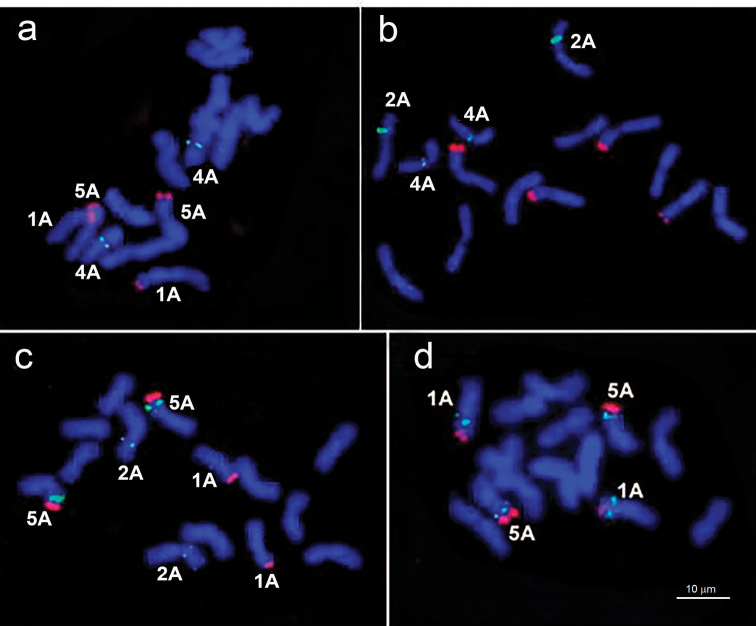
FISH with probes pTm30 (green signal) and pTa71 (red signal) on the chromosomes of diploid Triticum species: **a** *Triticum monococcum* MO1 **b** *Triticum boeoticum* BO3 **c** *Triticum boeoticum* BO14 and **d** *Triticum urartu* UR6.

The accessions of wild species *Triticum boeoticum* and *Triticum urartu* displayed polymorphism in the distribution of (GAA)n microsatellite on the chromosomes. One to three pTm30 sites per haploid genome can be detected in these two species (Table 2, Fig. 1). As a whole, (GAA)n can be detected in four positions, on the 1AS, 2AS, 5AS, and 4AL chromosomes of *Triticum boeoticum* and *Triticum urartu*. The (GAA)n site on the 1AS is observed only in *Triticum urartu*, being detectable in four of the seven examined accessions. Both *Triticum boeoticum* and *Triticum urartu* carry (GAA)n sites on 2AS and 5AS chromosomes; however, the microsatellite is detectable at a higher rate on the 5AS of *Triticum urartu* and the 2AS of *Triticum boeoticum*. (GAA)n site on the 2AS in one accession of *Triticum boeoticum* was heteromorphic between homologous chromosomes (Fig. 1c). Some accessions of *Triticum boeoticum* and *Triticum urartu* carry a (GAA)n site on the 4AL chromosome.

**Table 2.** Localization of pTm30 probe on the chromosomes of diploid Triticum species.

Chromosome (arm)	*Triticum boeoticum*^1^						*Triticum urartu*^1^							*Triticum monococcum*^2^
	BO2 IG1	BO3 IG2	BO9 IG1	BO12 IG2	BO14 IG1	BO19 IG2	UR1 IIG4	UR2 IIG4	UR3 IG3	UR4 IG1	UR5 IG2	UR6 IIG4	UR4 IIG4	
1A(S)								+			+	+	+	
2A(S)		+		+	+	+		+		+				
5A(S)	+			+	+		+	+			+	+	+	
4A(L)		+	+						+					+

1 Designation of accessions and their clustering into groups (I/II, superclusters and G, groups) are according to Konovalov et al. (2010).

2 Characteristic of five examined *Triticum monococcum* accessions; no pTm30 hybridization sites are detected for PI119423.

The domesticated species *Triticum monococcum* differs from the wild species by an almost complete lack of polymorphism in the distribution of pTm30 probe. The (GAA)n site in five of the six examined accessions is localized to the pericentromeric region of 4AL (Fig. 1a). No distinct hybridization sites of pTm30 have been found on chromosomes of accession PI119423.

### FISH analysis of Timopheevi wheats

**Figure 2.**
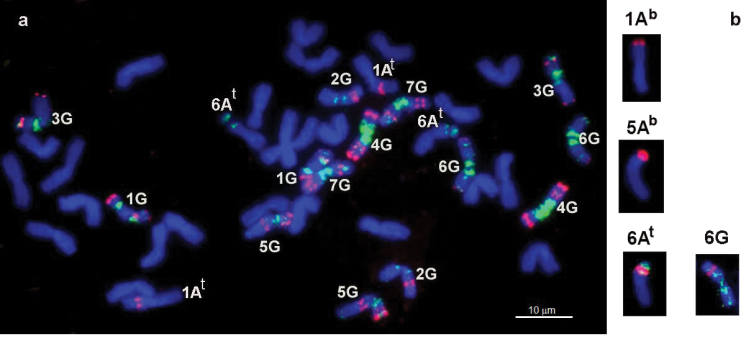
FISH with probe pTm30 (green signal) on the chromosomes of *Triticum zhukovskyi*: **a** red signal, pSc119.2 and **b** red signal, pTa71.

The examined accessions of *Triticum timopheevii* originated from the Zanduri population (Western Georgia), where the species *Triticum zhukovskyi* was first identified. *Triticum timopheevii* carries pSc119.2 signals predominantly on the G genome chromosomes and also on 1A^t^L and 5A^t^S; the pTa71 signals are present on 6A^t^S and 6GS chromosomes (Fig. 2). The pTm30 probe intensively hybridized to all G genome chromosomes and generates only one hybridization site on the chromosome 6A^t^S of the A^t ^genome (Fig. 2).

Similar results were obtained for hexaploid *Triticum zhukovskyi* (2*n* = 42, GGA^t^A^t^A^b^A^b^ genome), a natural amphiploid resulting from interspecific hybridization between *Triticum timopheevii* (2*n* = 28, GGA^t^A^t^ genome) and *Triticum monococcum* (2*n* = 14, AA genome). *Triticum zhukovskyi*, like *Triticum timopheevii*, carries the pTm30 site in the short arm of the 6A^t^ chromosome and pSc119.2 on 1A^t^L (Fig. 2). The remaining A^t^ genome chromosomes lack both pTm30 and pSc119.2 signals. The 45S RNA genes were detected on the 6A^t^S and 6GS chromosomes, as in *Triticum timopheevii*, and additionally on 1A^b^S and 5A^b^S, as in *Triticum monococcum* (Fig. 2). No pTm30 hybridization sites, which could have been donated by *Triticum monococcum*, have been detected.

### C-banding and FISH analysis of Emmer wheats

Since karyotyping of the A genome of polyploid wheats by FISH alone is not precise, Giemsa C-banding was also used in order to identify all chromosomes of *Triticum dicoccoides*, *Triticum durum*, and *Triticum aestivum*. In addition, the distribution pattern of probe pSc119.2 was considered, when identifying the chromosomes.

**Figure 3.**
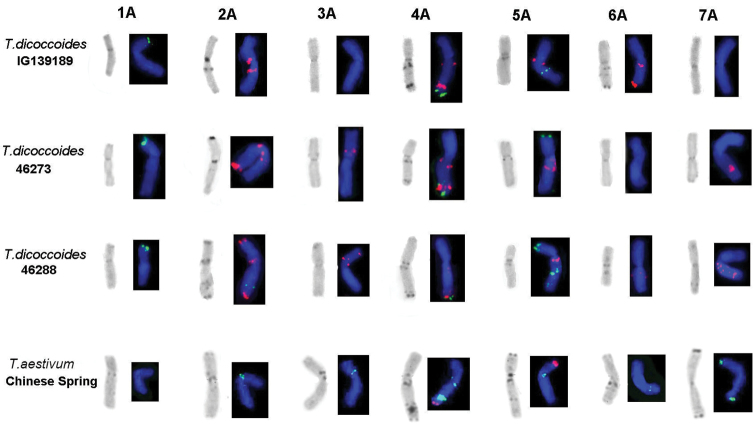
Localization of probe pTm30 on the chromosomes of Emmer wheats and C-banding. Accessions of *Triticum dicoccoides*: pTm30 (red) and pSc119.2 (green); accessions of *Triticum aestivum*: pTm30 (green) and pSc119.2 (red).

Among three *Triticum dicoccoides* accessions we identified the conserved pSc119.2 sites in subtelomeric regions of 1AS and 4AL chromosomes (Fig. 3), whereas polymorphic pSc119.2 sites were detected on chromosomes 5AS (subtelomeric localization), 5AL (intercalary localization), and 2AL (intercalary localization).

A comparative analysis of the A genome chromosomes of *Triticum dicoccoides* by FISH with pTm30 probe revealed both conserved and polymorphic (GAA)n signals (Fig. 3). The 1A lacked (GAA)n site during tetraploid formation, while up to two polymorphic (GAA)n loci were detectable on the 3A, 6A, and 7A chromosomes. The highest number of pTm30 hybridization sites was observed on the 2A and 4A chromosomes; of them, the pericentromeric site on 2AS and proximal and distal sites on 4AL were conserved. Chromosome 5AL carried a conserved (GAA)n site in the proximal region; no polymorphic blocks were detected. Note also that the localization of (GAA)n, as was expected, always coincided with the position of C-bands; however, for approximately 20% of the C-bands no corresponding (GAA)n regions have been detected.

The distribution of pTm30 hybridization sites on the A genome chromosomes of two hexaploid wheat cultivars, Chinese Spring and Saratovskaya 29, was similar (Fig. 3).

The absence of (GAA)n microsatellite on the 1A chromosome was the common feature for all *Triticum dicoccoides*, *Triticum durum*, and *Triticum aestivum* accessions. In addition, the conserved (GAA)n sites on *Triticum dicoccoides* chromosomes 2AS, 4AL, and 5AL were remained in *Triticum durum* and *Triticum aestivum*.

## Discussion

### (GAA)n as a cytogenetic marker for einkorn wheat

The goal of the search for cytogenetic markers for the wheat A genome dates back to the very first application of cytogenetic methods to analysis of chromosome structure and phylogeny of *Triticum* species. This was due to both a small number of C-bands detectable by Giemsa staining and the difficulties in FISH-based distinguishing between the A genome chromosomes. In particular, two cytogenetic markers, pSc119.2 and pAs1, are able to discriminate all B and D genomes chromosomes of Emmer wheat, but only three A genome chromosomes (Schneider et al. 2003). The pSc119.2 probe hybridized mainly to the B genome chromosomes of polyploid wheats belonging to Emmer evolutionary lineage, as has been demonstrated for hexaploid *Triticum aestivum* and tetraploid *Triticum durum* (Schneider et al. 2003; Kubaláková et al. 2005). In the A-genome of hexaploid wheat, the pSc119.2 hybridization sites were detected only on chromosomes 2AL, 4AL (subtelomeric localization), 5AS (subtelomeric localization), and 5AL (intercalary site). Note that different wheat cultivars display polymorphism in distribution of pSc119.2 probe on the A-genome chromosomes (Schneider et al. 2003). Involvement of additional probes—pTa71 (45S RNA) and pTa794 (5S RNA genes), localized to 1A (pTa71 and pTa794) and 5A (pTa794) chromosomes—failed to improve the resolution of this assay. The (GAA)n microsatellite, detected in all A genome chromosomes of common and durum wheat except for 1A (Fig. 3), is mainly used for chromosome sorting in polyploid wheats (Pedersen and Langridge 1997; Vrána et al. 2000; Kubaláková et al. 2005). However the direct application of this probe to phylogenetic studies of the A genome of polyploid wheats is hardly possible, because it gives only few minor signals compared to numerous major hybridization sites on the B and G genome chromosomes (Fig. 2; Kubaláková et al. 2005; Cuadrado et al. 2008).

The situation with chromosome identification in einkorn wheat is even more complex. The probes that are frequently used in molecular cytogenetic analysis of polyploid wheats, such as pSc119.2 and pAs1, either do not hybridize to einkorn chromosomes at all, or give few fuzzy signals (Megyeri et al. 2012, Danilova et al. 2012, I.G. Adonina, unpublished data). The rDNA markers, pTa71 and pTa794, produce conserved hybridization sites on the 1AS and 5AS chromosomes and fail to distinguish the einkorn genomes. An *Afa* probe, PCR-amplified from the genomic DNA of common wheat (Megyeri et al. 2012), may be the most promising for the study of chromosome reorganization of the A genomes. The *Afa* probe produces numerous hybridization sites; however, so far this has been demonstrated for only one accession of *Triticum monococcum*.

The distribution of (GAA)n microsatellite on chromosomes of the A genome diploid species has not been studied until recently. Dvorak (2009) wrote in his review referring to the works of Peacock et al. (1981) and Pedersen (et al. 1996) that (GAA)n sequence is absent in the diploid A genome donors of common wheat. However, karyotype analysis of individual *Triticum monococcum* and *Triticum urartu* accessions employing either (GAA)_9_ oligonucleotide or GAA fragments amplified by PCR from wheat genomic DNA has been recently reported (Danilova et al. 2012, Megyeri et al. 2012). Megyeri et al. (2012) studied one accession of *Triticum monococcum* and discovered two chromosomes with major hybridization sites of the (GAA)n probe in their distal and pericentromeric regions, which were identified as 2AS and 6AL, respectively, based on the distribution of *Afa* family and pTa71 probe. Danilova et al. (2012) identified the chromosomes more precisely using FLcDNAs and defined the chromosomes carrying the major sites as 2AS and 4AL in *Triticum monococcum* and 1AS in *Triticum urartu*. In addition, one to three minor (GAA)n sites were detected in two studied accessions of diploid species. So far, no publications describing the (GAA)n distribution on *Triticum boeoticum* chromosomes has been reported.

As has been demonstrated here, the pTm30 produces up to four major hybridization sites on the A genome chromosomes of diploid wheats (1AS, 2AS, 5AS, and 4AL), while any minor hybridization sites are undetectable. All four major hybridization sites are present in *Triticum urartu* only, and the site on 1AL is absent in *Triticum monococcum* and *Triticum boeoticum*. Interestingly, *Triticum urartu* accessions belonging to super-cluster II (*urartu*) mainly display two (GAA)n sites, on the 1AS and 5AS chromosomes, also carrying the 45S RNA genes. The major (GAA)n site on 1AS and minor site on 5AS have been also detected in the *Triticum urartu* accession by Danilova et al. (2012). An interesting fact has been obtained by FISH analysis of the three *Triticum urartu* accessions (UR3, UR4, and UR5), which were regarded as intermediate forms according to comparison of morphological and SSAP data (Tables 1 and 2). All three accessions differ in the distribution of (GAA)n microsatellite. However, UR5 accession is attributed to super-cluster II (*urartu*) according to pTm30 [(GAA)_56_] pattern, while UR3 and UR4 carry (GAA)n sites on the 2AS and 4AL chromosomes, which are mainly characteristic of *Triticum boeoticum* and *Triticum monococcum*. Cultivated einkorn *Triticum monococcum* displays the lowest polymorphism. We found pTm30 hybridization site on one chromosome pair only, designated 4AL.

Thus, it has been shown that the (GAA)n microsatellite can be used as marker for the 1AS, 2AS, 4AL, and 5AS chromosomes of einkorn wheat; however, it should be kept in mind that depending on species, the number of hybridization sites varies from zero to three in individual accessions. The (GAA)n site on chromosome 1AS is present only in *Triticum urartu*, while *Triticum boeoticum* and *Triticum monococcum* often carry (GAA)n site on the chromosome 4AL.

### Evolutionary reorganization of the A genomes in diploid and polyploid wheat species

The evolution of diploid and polyploid wheat species is known to be accompanied by reorganization of the genomes. At the diploid level, genome divergence occurs *via* accumulation of DNA mutations, amplifications/deletions of tandem repeats, proliferation of mobile elements, and, in some cases, chromosomal rearrangements (Devos et al. 1995, Salina et al. 2011, Fricano et al. 2014). Polyploid wheat displays a high level of chromosome rearrangements (Jiang and Gill 1994, Rodriguez et al. 2000, Salina et al. 2006a, Badaeva et al. 2007).

As any other tandem repeats, microsatellites frequently form large clusters on chromosomes, detectable with FISH. The polymorphism of satellite repeats most typically involve changes in the copy number, resulting in the appearance/elimination of large blocks of repeated sequences.

Study of the distribution of (GAA)n hybridization sites in diploid and polyploid wheats allows us to propose that several factors could have led to redistribution of regions housing this microsatellite. In particular, a decrease in the number of major microsatellite blocks in domesticated *Triticum monococcum* may only be a result genetic diversity shortage caused by bottleneck effect during domestication. Another important fact is that all studied accessions of polyploid wheat species *Triticum dicoccoides*, *Triticum durum*, *Triticum aestivum*, and *Triticum timopheevii* lack hybridization sites on the short arms of their 1A and 5A chromosomes (Fig. 3), which are characteristic of *Triticum urartu*, a putative donor of the A genome. The most likely reason for such event is the involvement of (GAA)n loci in reorganization of the nucleolus organizer region on the A genome chromosomes during formation and stabilization of primary allotetraploids which took place about 500 TYA. This resulted in total loss of 45S DNA locus and (GAA)n site on the 5AS as well as in the significant reduction in the number of 45S RNA gene copies (Jiang and Gill 1994) and the loss of (GAA)n site on the 1AS chromosome (Fig. 3). Hexaploid species *Triticum zhukovskyi* (genome GGA^t^A^t^A^b^A^b^) was formed about 60YA or more *via* the cross of *Triticum timopheevii* (genome GGA^t^A^t^) and *Triticum monococcum* (genome A^b^A^b^). According to our data, this species retained the 45S DNA loci on the 1A^b^S and 5A^b^S chromosomes, inherited from *Triticum monococcum*, however, the A^b^ genome chromosomes of *Triticum zhukovskyi* lacks (GAA)n sites observed in *Triticum monococcum*. This can be due to either the lack of such sites in the parental *Triticum monococcum* form, or elimination of (GAA)n loci over the course of amphiploidization, while the species was established.

As in the parental species *Triticum timopheevii*, *Triticum zhukovskyi* displays only one (GAA)n site in the short arm of chromosome 6A^t^, near the nucleolus organizer region. It is known that the T6AS/1GS translocation took place during *Triticum timopheevii* speciation (Jiang and Gill 1994). Thus, it is likely that the (GAA)n site on appeared a result of this translocation.

The (GAA)n sites of the einkorn wheat that are localized to more conserved chromosome regions, namely, pericentromeric regions of 2AS and 4AL, were inherited by all polyploid Emmer species (Fig. 3). No (GAA)n sites have been detected on the A^t^ genome homoeologous chromosomes of Timopheevi wheats, thereby confirming that their origin is independent from the Emmer group.

Thus, amplification and cloning of the long fragment of (GAA)n sequence from *Triticum monococcum* genome allowed us to obtain the new DNA probe for analysis of the A-genome chromosomes in diploid and polyploid wheat. An increased sequence length provides for higher probe stability, which enhances resolution of hybridization. Using a new probe we defined differences between A^b^ and A^u^ variants of the A-genomes, revealed variability of labeling patterns among *Triticum boeoticum* and *Triticum urartu* accessions, and significant shortage of polymorphism in *Triticum monococcum*, probably due to domestication. We suppose that distribution of (GAA)n sites in diploid and polyploid species reflects the chromosome reorganizations, mainly including the nucleolus organize region, that have taken place during the evolution of wild and domesticated species.
